# Oncology patients' and professional nurses' perceptions of important nurse caring behaviors

**DOI:** 10.1186/1472-6955-9-10

**Published:** 2010-06-15

**Authors:** Vahid Zamanzadeh, Roghaieh Azimzadeh, Azad Rahmani, Leila Valizadeh

**Affiliations:** 1 Department of Medical-Surgical Nursing, School of Nursing and Midwifery, Tabriz University of Medical Sciences, Tabriz, Iran; 2 Department of Child and Family Health, School of Nursing and Midwifery, Tabriz University of Medical Sciences, Tabriz, Iran

## Abstract

**Background:**

Caring is the essence of nursing. Caring to be meaningful needs to be based on mutual agreement between nurses and patients as to what constitutes nurse caring behaviors. As a result, healthcare professional can enhance patients' satisfaction of care by providing appropriate caring behavior. However, previous research that combined multiple types of patients, nurses and institutions demonstrated disagreement in prioritizing important behaviors. This paper reports a study that aimed at determining the caring behaviors which oncology patients and oncology nurses perceive to be the most important.

**Methods:**

This study is a comparative descriptive design that was conducted in an Iranian oncology centre. Convenience sampling was used to recruit 200 patients and 40 nurses to take part in the study. Data were collected over a period of 4 months in 2009 using the Caring Assessment Questionnaire, developed by Larson. Caring behaviors (n = 57) were ranked on a 5-point Likert-type scale and ordered in six subscales: "Being accessible", "Explains and facilitates", "Comforts", "Anticipates", "Trusting relationship", "Monitors and follows through". The data were analyzed using SPSS software version 13.0. The overall mean was calculated for each subscale to determine the rank distribution of the subscales. The nonparametric Mann-Whitney U test analysis of variables was used to compare patients' and nurses' scores on subscales.

**Results:**

The results demonstrate that both groups considered the same order of importance of caring, the high ranking of "Monitors and Follows through and "Being Accessible" and the low ranking of "Comforts" and "Trusting Relationships". Also, Patients only ranked "Being accessible" (*p = 0.04*) and "Explains and facilitates" (*p = 0.03*) higher than nurses.

**Conclusions:**

The oncology patients and nurses perceived highly physical aspects of caring and the results provide for nurses to be aware of the need, during their interactions with patients, to validate the effect their intended caring has upon patients. By so doing and with further refinement of the concept of caring for nursing in studies such as this, the practical aim of making patients feel cared for can be achieved.

## Background

Nursing as a professional discipline places the greatest demands specific to the development and refinement of the caring concept for nursing. By exploring the caring concept, within the boundaries of professional nursing practice, both the capabilities and constraints of caring relative to nursing, can be identified [1].

The study of human caring as an essential characteristic of nursing practice has gradually expanded from early definitional, philosophical and cultural writing on the meaning of caring [2]. Also, human caring is a universal phenomenon, but the expressions, processes and patterns vary among cultures [3]. This means culture and values affect our understanding of the concept of caring [4]. If so, it is important for scholars to ascertain nurses' and patients' perceptions of nursing care behaviors in different cultures.

On the other hand, according to Watson's theory, caring can be effectively demonstrated and practiced only interpersonally [5]. Thus the nurse-patient relationship forms the basis for nursing practice. This practical approach to the concept requires that both the patient's and the nurse's interpretations of caring be examined. To be meaningful, the caring of nursing must be based upon mutual agreement between nurses and patients as what constitutes nurse caring behaviors [1]. Nurses cannot be certain that their behavior is consistent with patients' perceptions of their care [6]. Also, nurses cannot assume that patients perceive caring efforts as they are intended. To avoid these problems, it is imperative that nurses validate with the patients that their care needs are being met [7,8].

In this regard, many researchers have asserted that there are two aspects of caring, expressive behaviors and instrumental activities. Expressive aspects of care involve providing emotional support to the patient through offers of fidelity, confidence, hope and emotional warmth. Instrumental aspects of care refer to substantial activities, such as giving bed bath and providing medical information, which promote physical comfort and cognitive coping [5].

Empirical studies on caring related to nursing have focused on nurse's perceptions of what constitutes caring for the patient, patient's perceptions of what is important in making them feel cared for, and comparisons of patient's and nurse's perceptions of what constitutes important nurse caring behaviors [9]. Many of these studies have generally demonstrated significant differences in patient's and nurse's perceptions of nurse caring behaviors [1,8,10-13].

The discrepancy in perceived importance of various caring behaviors between patients and nurses may result in patients' needs going unmet and patients' dissatisfaction with the care received [13].

Lack of perceptual agreement in previous studies has been attributed to samples that combined different types of patients and different types of hospital setting and thereby disregarded the possibility that perceptions of caring were influenced by the context in which the interaction occurred [7]. Therefore, the present study was designed to determine patients' and nurses' perceptions of nursing care behaviors from one subspecialty area and one oncology institution.

Oncology patients and oncology nurses were selected as the target population. Because the very nature of nurse interactions with oncology patients allow for frequent observation for the effect of their enacted behavior. Also, these patients, because of their diagnosis and treatment modalities, are often in frequent contact with professional nurses and may therefore be better able to formulate their perceptions of what nurse behaviors constitute caring to them [1].

It is evident that the majority of oncology patients' needs remain unmet in oncology wards [14], as nurses do not routinely detect and monitor the concerns of individuals with the diseases [15]. Also in our care system is less attention to oncology care [16] and there is not a comprehensive care plan based on the needs of the patients [17]. Therefore in our country, care of oncology patients has been encountered with several serious challenges that we should declare that there is a large gap with international standards which must be filled [18].

In this respect, such studies increases knowledge on the caring nature of oncology nursing, can facilitate a plan of nursing care based on priority caring behaviors identified by oncology patients and identifies the degree to which this sample of oncology patients and nurses agree on priority caring behaviors [7]. There is currently a considerable emphasis on the provision of patient-centered care in all aspects of health care [19]. What is most important is to make clear what influences patient satisfaction to improve quality of nursing care. Some effective factors on patients' satisfaction are "socio-demographic background of the patients, patients' expectations regarding nursing care, interpersonal relations between nurse and patient, nurses' medical-technical competence ..." [20].In the field of nursing, the most widely accepted definition is that of Risser according to which patients' satisfaction with nursing care is the degree of convergence between the expectations patients have of ideal care, and their perception of the care they really get [21]. It would be better to say that there is a relationship between nurses' caring behaviors and patients' satisfaction as having a high correlation [22]. Healthcare professionals can enhance patients' satisfaction of care by providing appropriate caring behavior [13].

In spite of that, there aren't adequate studies on nurses' and patients' perceptions in regard to nursing care of these patients in African and Asian countries including Iran. As such, it is important to investigate the perceived importance of nurse caring behaviors of oncology patients and their nurses.

So the aim of this study was to determine the caring behaviors which oncology patients and oncology nurses perceive to be most important in making patients feel cared for.

## Methods

### Design

A comparative descriptive design was used in this study. This design is used to describe and examine differences in variables in two or more groups that occur naturally in the setting [23]. This approach is appropriate for this study of examining oncology patients' and oncology nurses' perceptions of nurse caring behaviors.

### Setting

The target population for this study was oncology patients and oncology nurses from a comprehensive oncology center in Tabriz, Iran, where these patients are treated and is a center that covers all of oncology patients in Northwest of Iran which is one of the greatest areas covered.

### Participants

Nurses, who had worked on the two oncology wards for at least six months, were eligible to participate. All 40 nurses who were asked to take part in the study agreed to participate (response rate 100%).

We selected the participants by using convenience sampling. All potential patients were individually asked to participate during the study The inclusion criteria consisted of age ≥ 15 years, a cancer diagnosis known to the patient, ability to answer questions (physically or mentally) and to understand and speak Azeri or Persian and having at least one prior hospitalization. Of a total of 652 patients, 210 were selected by these criteria; however 10 persons did not want to participate mainly because of symptoms and tiredness. Therefore the final sample included 200 patients. Initially for parametric estimate of sample size in patients, indicated that 141 patients would be sufficient for our study (p = 0.9, α = 0.05, M_1 _= 4.03, M_2 _= 3.84, SD_1 _= 0.53, SD_2 _= 0.45).

### Data collection

Data were collected using a questionnaire over a period of 4 months (between May and August, 2009). The research assistant reviewed the nurses list and the inpatients admission list, three times per week and then identified potential study participants. Eligible participants were approached individually with an explanation of the study. Then the instrument pack (containing the consent form, directions for doing the scoring, a demographic data sheet and main questionnaire of caring) were reviewed with the study participants. Questions were answered as needed.

### The Caring Assessment Questionnaire (Care-Q)

The Care-Q was developed by Larson (1981) for use with Q-methodology (forced-choice format with quasi-normal distribution) to measure the perceived importance of nurse caring behaviors of oncology patients and oncology nurses. This Care-Q is the most frequently used instrument for assessing caring in the world and therefore the most appropriate instrument for international comparison [4]. The original questionnaire consisted of 50 caring behaviors that were categorized into the following 6 subscales. "Being accessible" (6 items), "Explains and facilitates" (6 items), "Comforts" (9 items), "Anticipates" (5 items), "Trusting relationship" (16 items), "Monitors and follows through" (8 items). In this study, we have converted the Care-Q forced response format to a Likert-type free rating scale. Scores assigned to each item were between 1 and 5 points, grading from the least important (1) to the most important (5). The reason for this is that in our pilot study was determined that besides the lengthy time required completing the Care-Q with forced-choice format, another problem is that some participants did not sort the cards according to the directions of that. Also, Andrews et al. highlighted this issue [24]. The results of Widmark-Petersson et al. demonstrated that forced-choice vs. free-choice response formats did not affect patients' or nurses' answers [25].

In the current study, the Care-Q was translated to Persian and was verified by content validity. So first the psychometric properties of the Care-Q related to validity and reliability were assessed. Content validity was evaluated by different expert panels (2 oncologists, 4 nurses, and 6 nurse educators) and some alternations were made based on their suggestions. Internal consistency reliability was determined by using the study sample responses to calculate Cronbach's α (alpha) for each of the 6 subscales. Results show internal consistency reliabilities of 0.93 for total items and from 0.61 to 0.80 for the 6 subscales using 40 oncology patients. Also in 10 nurses' responses, Results show internal consistency reliabilities of 0.94 for total items and from 0.41 to 0.84 for the 6 subscales.

For cultural reasons, only three items were added to this new version of Care-Q and four items of original ones that conveyed different concepts were separated into two items. As a result, the Care-Q consists of 57 caring behaviors that were ordered in the following 6 subscales: "Being accessible" (6 items), "Explains and facilitates" (9 items), "Comforts" (11 items), "Anticipates" (5 items), "Trusting relationship" (18 items), "Monitors and follows through" (8 items).

This version of instrument was pilot tested with 15 patients and 5 nurses. Several minor comments were submitted and some were used for revision, such as suggestions about the better scoring of the instrument. Finally patients were instructed to score the items according to the following question "In order to make you feel cared for, how important is it that the nurses...?" Also the nurses were asked the corresponding question of " how important they thought each item was in order to make the patients feel cared for". Nurses and patients were asked to complete the Care-Q without assistance, but if a patient was unable to complete the questionnaire without assistance, the research assistant read the questionnaire items to the patients and then recorded the patients' answers on the questionnaire. Each questionnaire took 15-20 minutes to complete. All participants were asked to fill out a background data sheet.

## Ethical considerations

Before beginning the study, approval was obtained from the Ethical Committee of Tabriz University of Medical Science. The research assistant met with each participant explained the purpose of the study and the instrument packet materials. Moreover, written and oral informed consent to participate in the study were obtained from each participant who agreed to complete the instrument (of course, for patients under 18 years, we were obtained consensus by their parents). It emphasized that participation was voluntary and subjects were assured of confidentiality.

## Data Analysis

The data were analyzed using SPSS software version 13.0 and each questionnaire item was first coded for statistical analysis from 1 for the least important to 5 for the most important and then mean scores and standard deviations were calculated to find the most important nurse caring behaviors. The overall mean for each individual was calculated for each subscale to determine the rank distribution of the subscales. The nonparametric Mann-Whitney U test analysis of variables was used to compare patients' and nurses' scores on subscales and for individual behaviors. The level of significance was set at *p *< 0.05.

## Results

### a) Background Variable

Nurse participants (n = 40) ranged in age from 26 to 52 (a mean age of 36.7, SD = 6.6) years; most (92.5%) were women, and two-thirds (65%) were married. Of these, one person (2.5%) was nurse practitioner with a level of education in MSN (Master of Science in Nursing); thirty-four (85%) were Registered nurses with a level of education in BSN (Bachelor of Science in Nursing) and four (12.5%) were practical nurses at diploma level. The mean length of clinical experience was 12.2 (SD = 6.92) years, and the mean length of providing oncology care was 8.01 (SD = 5.81) years, indicating that the vast majority of nurse participants were very experienced in caring for oncology patients.

Also, the study patient participants (n = 200) ranged in age from 15 to 85 (a mean age of 44.7, SD = 17.75) years. One hundred-three of the patients (51.5%) were women and 97 (48.5%) were men and the majority (77.5%) were married. The level of education in patients was uneducated 28.5% (n = 57), primary education 39% (n = 78), secondary education 23% (n = 46) and tertiary education 8.5% (n = 17). For these patients, numbers of priori hospitalizations in this center were 78% (n = 156) lower than five, 15.5% (n = 31) between six and ten, 6.5% (n = 13) upper than eleven.

The original diagnosis of cancer consisted of systems such as: hematological 31.5% (n = 63), digestive 27% (n = 54), Lymphatic 13.5% (n = 27), urogenital 7% (n = 14), respiratory 6% (n = 12), musculoskeletal 6% (n = 12) and other systems 8.5% (n = 17).

### b) Care-Q

#### b-1) Subscale analysis

Mean scores were calculated for each of the Care-Q six subscales for the patients' and nurses' groups which ranged from 3.95 to 4.32 and 3.83 to 4.42, respectively. The ranking of the six subscales in order of importance from patients' and nurses' groups are presented in Table 1. The results showed patients and nurses perceived "Monitors and follows through", "Being accessible" as the most important and "Comforts", "Trusting relationship" as the least important subscale. Indeed, there are the same ranking for patients and nurses. Also these mean scores showed that patients gave higher mean values than did nurses to a large number of subscales. This indicates that patients, to a greater extent than nurses, consider several Care-Q dimensions to be of a very high importance. For two groups mean scores on the subscales were compared with Mann-Whitney *U *tests. There were significant differences only among 2 of the 6 subscales. Patients ranked "Being accessible" (*p = 0.04*) and "Explains and facilitates" (*p = 0.03*) higher than nurses.

**Table 1:** Mean Values of Patients and Nurses on Caring Subscales in Rank Order and Their Comparison

Care-Q Subscales	Patients (n = 200)			Nurse (n = 40)			**U**. ^**b**^	Z	***P***. ^**c**^
	Mean ± SD^*^	ranking	(CI^**^)	Mean ± SD	ranking	(CI)			
"Monitors and follows through" (8 items)	4.32 ± 0.41	1	(4.26-4.38)	4.42 ± 0.44	1	(4.27-4/56)	3355.50	-1.615	0.106
"Being accessible" (6 items)	4.32 ± 0.44	2	(4.25-4.38)	4.19 ± 0.37	2	(4.07-4.31)	3194.50	-2.024	0.043*
"Anticipates" (5 items)	4.19 ± 0.57	3	(4.11-4.27)	4.12 ± 0.49	3	(3.96-4.27)	3659.00	-0.857	0.391
"Explains and facilitates" (9 items)	4.17 ± 0.49	4	(4.10-4.24)	4.00 ± 0.53	4	(3.83-4.17)	3133.00	-2.168	0.030 *
"Comforts" (11 items)	4.06 ± 0.50	5	(4.00-4.13)	3.96 ± 0.51	5	(3.79-4.12)	3428.50	-1.429	0.153
"Trusting relationship" (18 items)	3.95 ± 0.45	6	(3.89-4.02)	3.83 ± 0.56	6	(3.65-4.01)	3726.00	-0.684	0.494

*. Highest possible mean = 5, lowest possible mean = 1 **. Confidence Interval

b. Mann-Whitney *U *test c. *p < 0.05*

#### b-2) Item analysis

Also mean scores were calculated for each of the 57 Care-Q items for the two groups. The maximum possible score was 5 and the minimum was 1. Mean scores for items ranged from 2.88 to 4.83 for patients and 2.82 to 4.90 for nurses.

Items were ranked in order of importance by the participants. The 10 most important Care-Q behaviors rated by patients and nurses are presented in Table 2 and 3, respectively. A comparison of these top 10 Care-Q items between patients' and nurses' perceptions revealed similarities as well as differences. Patients and nurses agreed on 5 out of the 10 most important items. The following five items (common items) were ranked among the top 10 by both patients and nurses include: "Gives the patients' treatments and medications on time"; "Knows how to give shots, I.V.s", etc (these common items are showed in Tables 2-3 with bold words). Interestingly again, the first two items were the same for both groups. These results demonstrated a basic harmony between the two groups with respect to the priorities of care (in the two groups' highest ranked items, the additional items are showed in Tables 2-3 without being bold).

**Table 2:** Patients' Rankings of the 10 Most Important Caring Behaviors and Their Comparison to Nurses' (n = 200)

Care-Q items	Subscale a	Patients		**U**. ^**b**^	Z	***P***. ^**c**^	Nurses	
		(Mean ± SD)	Ranking				(Mean ± SD)	Ranking
**1- "Gives the patients' treatments and ** **medications on time"**	**AC**	**(4.83 ± 0.43)**	**1**	**3794.0**	**-0.85**	**0.39**	**(4.90 ± 0.30)**	**1**
**2- "Knows how to give shots, I.V.s, etc, and ** **how to manage equipment like ** **I.V.s, suction machines, etc"**	**M&F**	**(4.77 ± 0.47)**	**2**	**3952.5**	**-0.16**	**0.86**	**(4.79 ± 0.40)**	**2**
**3- "Carry out therapeutic care skillfully ** **to make at least suffering for patients"**	**C**	**(4.68 ± 0.52)**	**3**	**3438.0**	**-1.73**	**0.08**	**(4.52 ± 0.59)**	**6**
4- "Checks on the patients frequently"	AC	(4.66 ± 0.54)	4	2439.5	-4.58	0.00*	(4.22**± 0.61**)	21
5- "Is cheerful"	C	(4.65 ± 0.67)	5	1968.5	-6.05	0.00*	(3.93**±**0.80)	35
6- "Is patient even with 'difficult' patients"	C	(4.64 ± 0.69)	6	2404.5	-4.79	0.00*	(4.16**± 0.73**)	25
7- "Is well organized"	M&F	(4.64 ± 0.61)	7	3230.0	-2.33	0.02*	(4.39**±**0.80)	12
**8- "Is calm"**	**M&F**	**(4.57 ± 0.66)**	**8**	**3564.5**	**-1.27**	**0.20**	**(4.43 ± 0.74)**	**8**
9- "Gives a quick response to the patients' call"	AC	(4.57 ± 0.65)	9	3169.0	-2.40	0.01*	(4.28**±**0.78)	15
**10- "Is perceptive of the patients' needs ** **and plans and acts accordingly"**	**AN**	**(4.55 ± 0.65)**	**10**	**3543.0**	**-1.32**	**0.18**	**(4.42 ± 0.67)**	**9**

a. AC = "Being accessible"; M&F = "Monitors and follows through"; C = "Comforts"; AN = "Anticipates";

b. Mann-Whitney *U *test c. p < 0.05 *. Confidence Interval

**Table 3:** Nurses' Rankings of the 10 Most Important Caring Behaviors and Their Comparison to Patients' (n = 40)

Care-Q items	**Subscale**. ^**a**^	Nurses		**U**. ^**b**^	Z	***P***. ^**c**^	Patients	
		(Mean ± SD)	Ranking				(Mean ± SD)	Ranking
**1- "Gives the patients' treatments and ** **medications on time"**	**AC**	**(4.90 ± 0.30)**	**1**	**3794.0**	**-0.85**	**0.39**	**(4.83 ± 0.43)**	**1**
**2- "Knows how to give shots, I.V.s, etc, ** **and how to manage equipment ** **like I.V.s, suction machines, etc"**	**M&F**	**(4.79 ± 0.40)**	**2**	**3952.5**	**-0.16**	**0.86**	**(4.77 ± 0.47)**	**2**
3- "Treats with information of the patients confidentially"	TR	(4.66 ± 0.61)	3	3278.5	-2.04	0.04*	(4.36 ± 0.84)	13
4- "Knows when to call the doctor"	M&F	(4.65 ± 0.47)	4	2562.5	-3.89	0.00*	(4.14**± 0.81)**	29
5-"Is professional in appearance, wears appropriate identifiable clothing and identification"	M&F	(4.52 ± 0.81)	5	2779.0	-3.28	0.00*	(4.09 ± 0.93)	32
**6- "Carry out therapeutic care skillfully ** **to make at least suffering for patients"**	**C**	**(4.52 ± 0.59)**	**6**	**3438.0**	**-1.73**	**0.08**	**(4.68 ± 0.52)**	**3**
7- "Tells the patients in understandable language"	E&F	(4.47 ± 0.55)	7	3652.5	-0.96	0.33	(4.32**± 0.72**)	21
**8- "Is calm"**	**M&F**	**(4.43 ± 0.74)**	**8**	**3564.5**	**-1.27**	**0.20**	**(4.57 ± 0.66)**	**8**
**9- "Is perceptive of the patients' needs ** **and plans and acts accordingly"**	**AN**	**(4.42 ± 0.67)**	**9**	**3543.0**	**-1.32**	**0.18**	**(4.55 ± 0.65)**	**10**
10- "Anticipates that the 'first time' are the hardest and pays special attention to the patients during these times"	AN	(4.40 ± 0.67)	10	3783.5	-0.59	0.55	(4.30**± 0.76**)	22

^a.^AC = "Being accessible"; M&F = "Monitors and follows through"; E&F = "Explains and facilitates"; C = "Comforts"; TR = "Trusting relationship"; AN = "Anticipates";

b. Mann-Whitney *U *test c. p < 0.05 *. Confidence Interval

Also, there were significant differences (*p < 0.05*) between patients and nurses within the top 10 caring behaviors (see Tables 2-3). These results indicated that nurses more than patients value to the subscale "Monitors and follows through". In spite of that, patients more than nurses value the subscales "Being accessible" and "Comforts". These differences may demonstrate that oncology patients believe that nurses should be accessible and promote comfort for them, because of their situations.

Furthermore, in total there were significant differences between patients and nurses for 22 out of the 57 (38% or a little bit more of one-third) individual Care-Q items. The items that patients ranked significantly higher than nurses are from the following subscales: "Being accessible" (2), "Explains and facilitates" (3), "Comforts" (3), "Anticipates" (1), "Trusting relationship" (6) and "Monitors and follows through" (1). Nurses gave higher scores to behaviors belonging to the following subscales: "Comforts" (2), "Trusting relationship" (1) and "Monitors and follows through" (3). These results also indicated that unlike nurses, patients markedly had given higher mean value for all dimensions of caring and like that nurses perform their duty competently.

## Discussion

This study demonstrated marked concordance between oncology patients' and nurses' views in prioritizing how important different nurse caring behaviors are considered to be "important in making you/the patient feel cared for".

Both the nurses and patients perceived behaviors determining nurses' competency in professional knowledge and care surveillance or practical behaviors to be more important than psycho-social skills.

The findings differ from a large number of earlier studies which showed that patients and nurses did not concur on the importance of caring behaviors [1,8,10,13]. These studies demonstrated that caregivers tend to stress the more qualitative dimensions of care and underrate physical care issues, which are perceived by patients as more essential. However, it seems that there are exceptions to this pattern because other study results clearly indicate that clinical skills are still valued and respected by a proportion of nurses. For example, Keane et al's and Azizzadeh et al's studies explored perceptions of caring by nurses and patients in rehabilitation and medical-surgical settings, respectively. Making use of the Care-Q instrument, they found that both groups viewed competent clinical expertise as the most important component of a nurse-patient caring interaction [26,27]. Also, Dowling found that nurses' technical skills were alluded to by both nurses and patients interviewed as a contributing factor to the closeness of their relationship. When the patient trusted the nurse's competence with regard to their technical skills, they wanted that nurse to care for them [28]. In a recent theoretical account on caring, researcher supported the notion that helping patients with big (e.g. pain relief) or little (e.g. hair dressing) things regarding their physical care is an important element of the caring process [29].

In the present study, the concordance of perceptions may be due to the fact that unlike others in this study, patients with the same disease and nurses working in the same unit/setting were surveyed. The oncology patients and nurses may have been had a long-term interactions with each other, so that they may have established more consistent perceptions regarding the importance of caring behaviors [13].

Interestingly both groups in our study ranked the six subscales in the same order. This unique finding, in comparison to other studies [1,11,10,8,13] showed that this sample of oncology patients and nurses were in strong agreement in priority behaviors.

For patients, the high ranking of "Monitors and Follows through" and "Being Accessible" and the low ranking of "Comforts" and "Trusting Relationships", is in accordance with previous results [1,6,11-13,30]. However, the findings on nurses are in contrast to earlier studies in which "Monitors and Follows through" [1,11,10] or "Being Accessible" [8,11] were as the least important subscales, but "Comforts" [1,10,11] was as the most important ones. These are in similar to studies [1,10,8,13] in which "Trusting relationship" was as the least important subscale.

A possible explanation of these differences is that nurses in the present wards monitored highly advanced treatments for cancer diagnosis and therefore may have considered this Care-Q subscale more important than nurses in earlier studies did [8].

Patients gave a significantly higher mean value than did nurses to "Being Accessible" subscale, which is in contrast to previous studies [10] in which nurses gave higher values than did patients to this subscale. As suggested earlier for "Monitors and Follows through", this finding may also depend on the advanced treatments of these patients.

Also compared to similar studies [8,10,12] in the literature, this study did show significantly higher ranking of the importance "Explain and facilitates" by patients than by nurses. For this, the large numbers of studies clearly show the importance of the fact that caregivers should have advanced knowledge of how to give detailed explanations to patients and to plan the required professional activities. In support of this, Carlberg and Tibblin found that the most important factor for a high degree of patient satisfaction was sufficient and understandable information [31].

The findings of this study indicated that patients and nurses agreed on 5 of the 10 most important items. Patients scored "Gives the patients' treatments and medications on times", "Knows how to give shots..." and "Checks on the patients frequently"...as the 10 top caring behaviors. Also nurses ranked "Gives the patients' treatments and medications on times", "Knows how to give shots..." and "knows when to call the doctor"... as the most significant ones (Tables 2-3). The first two items of ranking were in the same order for both groups. These items included the "Monitors and Follows through" and "Being Accessible" subscales and is related to physical aspects of nursing care or nurse competence. Indeed, these results presenting caring as the performance of basic nursing care activities are in accordance with Maslow's hierarchy of needs and the life-saving purpose of professional actions [32]. Also nurses should demonstrate their technical skills and scientific knowledge to meet basic needs of the patients before they proceed to address the emotional and affective aspects of caring [33] .

This study also found that there are some agreements and disagreements between the findings of this study and previous ones. Our findings are in agreement with those studies [1,6,13,27] in which patients assigned "Gives the patients' treatments and medications on times", "Knows how to give shots..." and "Checks on the patients frequently" as among the 10 most important caring behaviors. However, nurses assigned "Gives the patients' treatments and medications on times" and "Knows how to give shots..." only in Azizzadeh et al's and Chang et al's studies [13,27], and "Treats with information of the patients confidentially" in Larson and Larson et al's studies as 10 top ones [1,10].

On the other hand, our results for nurses differ from those studies [1,10-12] in which "listen to the patient, "talk to the patient" and "touch the patient" were among the 10 most important caring behaviors. These behaviors were not among the 10 top caring behaviors in our study.

These findings support the Leininger's beliefs that asserted human caring is a universal phenomenon, but the expressions, processes and patterns vary among cultures" (p. 11) or caring behaviors and functions vary with social structure features of any designed culture. Indeed, to provide therapeutic nursing care, the nurse should have knowledge of caring values, beliefs and practices of the patients. Patients have the right to be "cared for" in a way that indicates respect for their cultural diversity [3].

To sum up, mean values for Care-Q subscales and individual behaviors demonstrated concordances between patients' and nurses' priorities for four of six subscales and almost two-third behaviors. Only a few significant differences were found. Taken together, the results suggest that patients and nurses do agree strongly on the importance of various caring behaviors.

The results of the current study, in summary, indicate that nurses do know their patients well enough to judge what aspects of caring the latter consider important in order to feel well cared for. The interesting results of the study suggest that nurses have obtained a lot of information from the individual patients' concerning problems and needs. It is possible that nurses ask about these matters and nurses want to use their time to benefit the patient in the best possible way. Furthermore, it is time to listen to the patients' views and perspectives.

Of course, the findings of the present study need to be considered in light of several methodological limitations. The small nurse sample produced unequal sample sizes to compare the two groups. Also, a convenience sampling for patients and nurses from two wards of one setting were used; therefore the findings cannot be generalized to other oncology patients or institutes. Also, in this study for some subscales of the instrument, Cronbach's α (alpha) was calculated low (0.61 and 0.41) and so this make the reliability issue as a limitation to the generalization of the study results.

Furthermore, most patients pointed out that the many numbers of the instrument items (n = 57) needed lengthy time for completion it, as such this was accomplished with some patients in two or three different periods. The majority of the respondents tended to nominate the top two values of the 5-point Likert scale for most scale items. This makes it difficult to make a true distinction between the relative importance of the items and the subscales. It may be that the concept of caring is an integrated concept which is hard to divide into subsets.

## Conclusions

The current study comprising oncology patients and nurses determined that perceptions of caring were very highly concordant in this sample. Increasingly high extent agreement between patients and nurses as to the importance of caring behaviors could have great potential for improving the quality of nursing care [13]. Furthermore, the "Being accessible" and "Explains and facilitates" subscales were more value by patients than nurses, so it is needed that nurses notice this issue in clinical work. Also, both the oncology patients and nurses perceived highly physical aspects of caring. However, for delivering holistic care, oncology nurses must value affective/emotional aspect of caring, too.

The results from this study provide improvement implications for the care of patients, such as some concrete information on what behaviors the patient would like to experience. This information can be given to nurses in order to enhance the way they provide care to patients. Also programs may be developed in order to help nurses meet the caring expectations of patients [34]. In fact, the greatest implication for practice from this study is for nurses to be aware of the need, during their interactions with patients, to validate the effect their intended caring has upon patients. By so doing and in conjunction with further refinement of the concept of caring for nursing in studies such as this, the practical aim of making patients feel cared for can eventually be achieved on prescriptive basis.

The perceptions of the most important caring behaviors by oncology patients and oncology nurses need further investigation to ensure a truer understanding of what is needed to develop even more agreement on caring priorities. It is recommended that further qualitative research be carried out to ascertain what caring behaviors are perceived as important in an oncology setting. Also more work is required into examining the pragmatics of using a quantitative instrument with oncology respondents. Methods such as Likert-scales may confuse some patients, so we need to develop more user friendly options.

This study only investigates the meaning of caring from the perspectives of oncology patients in one city in Iran. Further exploration in different clinical settings is needed in order to understand Iranian culture-based specificities about caring in nursing.

## Competing interests

The authors declare that they have no competing interests.

## Authors' contributions

VZ, RA and AR were responsible for the study conception and design; RA performed the data collection; VZ, RA and LV performed the data analysis; RA was responsible for the drafting of the manuscript; VZ, AR and LV made critical revisions to the paper for important intellectual content; VZ supervised the study.

## Pre-publication history

The pre-publication history for this paper can be accessed here:

http://www.biomedcentral.com/1472-6955/9/10/prepub
